# Acute myeloid leukemia cell membrane-coated nanoparticles for cancer vaccination immunotherapy

**DOI:** 10.1038/s41375-021-01432-w

**Published:** 2021-11-29

**Authors:** Daniel T. Johnson, Jiarong Zhou, Ashley V. Kroll, Ronnie H. Fang, Ming Yan, Crystal Xiao, Xiufen Chen, Justin Kline, Liangfang Zhang, Dong-Er Zhang

**Affiliations:** 1 Moores Cancer Center, University of California San Diego, La Jolla, CA USA; 2 Biological Sciences Graduate Program, University of California San Diego, La Jolla, CA USA; 3 Division of Biological Sciences, University of California San Diego, La Jolla, CA USA; 4 Department of NanoEngineering, University of California San Diego, La Jolla, CA USA; 5 Section of Hematology/Oncology, University of Chicago, Chicago, IL USA; 6 Department of Pathology, University of California San Diego, La Jolla, CA USA

**Keywords:** Biotechnology, Cancer immunotherapy, Acute myeloid leukaemia, Preclinical research, Acute myeloid leukaemia

## Abstract

Cancer vaccines are promising treatments to prevent relapse after chemotherapy in acute myeloid leukemia (AML) patients, particularly for those who cannot tolerate intensive consolidation therapies. Here, we report the development of an AML cell membrane-coated nanoparticle (AMCNP) vaccine platform, in which immune-stimulatory adjuvant-loaded nanoparticles are coated with leukemic cell membrane material. This AMCNP vaccination strategy stimulates leukemia-specific immune responses by co-delivering membrane-associated antigens along with adjuvants to antigen-presenting cells. To demonstrate that this AMCNP vaccine enhances leukemia-specific antigen presentation and T cell responses, we modified a murine AML cell line to express membrane-bound chicken ovalbumin as a model antigen. AMCNPs were efficiently acquired by antigen-presenting cells in vitro and in vivo and stimulated antigen cross-presentation. Vaccination with AMCNPs significantly enhanced antigen-specific T cell expansion and effector function compared with control vaccines. Prophylactic vaccination with AMCNPs enhanced cellular immunity and protected against AML challenge. Moreover, in an AML post-remission vaccination model, AMCNP vaccination significantly enhanced survival in comparison to vaccination with whole leukemia cell lysates. Collectively, AMCNPs retained AML-specific antigens, elicited enhanced antigen-specific immune responses, and provided therapeutic benefit against AML challenge.

## Introduction

Acute myeloid leukemia (AML) continues to be associated with a poor prognosis, with an overall 5-year survival of ~29% [1]. Although many patients achieve complete remission after initial induction chemotherapy [2], there remains a high rate of relapse due to the persistence of a small number of therapy-resistant leukemic cells, termed minimal residual disease (MRD) [3, 4]. The current standard treatment strategy to clear MRD is intensive consolidation therapy with allogeneic hematopoietic stem cell transplantation (HSCT) [5]. Unfortunately, HSCT carries significant morbidity and is unsuited for older or less healthy patients [5, 6]. While the recent approval of various targeted AML therapies has improved survival in certain patient subgroups, relapse after initial therapy remains a large problem [7, 8]. Thus, there is a great need for alternative tolerable consolidation therapies that target MRD to delay or prevent relapse.

Cancer immunotherapy has revolutionized the treatment of a growing number of human cancers. Although therapeutic cancer vaccines have not been successful in subjects with large tumor burden, they may be useful as consolidation therapy for AML in the MRD state [9, 10]. The underlying strategy of cancer vaccines is to enhance the presentation of cancer-specific or cancer-associated antigens by activated professional antigen-presenting cells (APCs) to induce effective and durable T cell immunity [10]. However, presentation of leukemia antigens by quiescent APCs is insufficient to stimulate functional leukemia-specific T cell responses, and rather can lead to a T cell tolerant state [11]. The efficient activation of APCs in the context of cancer vaccination requires the co-administration of antigens with immunostimulatory adjuvants [12–14]. Thus, a key component of AML vaccination immunotherapies is the effective delivery of both AML-associated antigens and immunostimulatory adjuvants to APCs [9, 10].

Recent studies have highlighted the promise of “personalized” anticancer vaccination with identification of patient-specific neoantigens [15–19]. Because AML is a heterogeneous disease, AML vaccination strategies should ideally induce a multi-antigenic immune response. Clinical trials have recently shown the feasibility and promise of personalized and multi-antigenic AML vaccines using various methods, including pulsing ex vivo antigen-presenting dendritic cells (DCs) with autologous apoptotic AML blasts/lysates as the antigen source [20–22], generating ex vivo DCs differentiated directly from AML blasts [23–25], or fusing ex vivo DCs with autologous AML blasts [26]. These strategies have proven to be well tolerated [20–26], elicit AML-associated T cell responses [21–24, 26], and protect against disease relapse [25, 26]. While ex vivo strategies benefit from easily providing DCs with adjuvants and antigens, they face challenges associated with generating sufficient DCs, homing ex vivo vaccinated DCs to lymph nodes, and maintaining active DCs in culture [27]. These challenges may potentially be overcome by in vivo vaccination strategies. Indeed, in vivo administration of allogeneic or autologous AML whole cell vaccines induced leukemia-associated immunity and prolonged overall survival, although responses have varied among patients [28–30]. The promising results of these vaccines may be improved upon through the development of strategies to simultaneously deliver immunostimulatory adjuvants and a broad array of leukemic antigens to individual APCs in vivo.

Cell membrane coating nanotechnology is an emerging approach for developing multifunctional biomimetic nanoformulations consisting of a synthetic nanoparticle core coated in a layer of natural cell membrane [31]. Leveraging this technology, it has been demonstrated there may be tremendous benefits in the nanodelivery of antigen-rich cancer cell membrane material for anticancer vaccination [32]. A cancer cell membrane-coated nanovaccine employing an adjuvant-loaded polymeric core was recently developed to treat melanoma [33]. As a prophylaxis, the nanovaccine protected a majority of vaccinated mice from developing tumors in a lowly immunogenic B16F10 model. When used in combination with immune checkpoint inhibitors, effective control of tumor growth was also achieved in a therapeutic setting. This work highlighted the potential of leveraging a cell membrane-coated nanovaccine to generate systemic antitumor immunity, which is an even more important consideration for blood cell malignancies such as leukemia. AML is an attractive target for the application of this technology because suitable numbers of malignant blasts can be harvested and enriched from bone marrow aspirates or apheresis as a source of antigen-rich membrane material [22–24, 26, 28, 29].

Here, we developed an AML cell membrane-coated nanoparticle (AMCNP) vaccine to treat liquid tumors in a clinically relevant setting, targeting MRD and aiming to prevent relapse. AMCNPs are adjuvant-loaded nanoparticles wrapped with antigen-rich leukemic cell membrane material and were readily deliverable to APCs in vitro and in vivo. Upon AMCNP acquisition, APCs presented leukemia membrane-associated antigens. Furthermore, vaccination with AMCNPs led to enhanced leukemia-specific and leukemia-associated antigen-specific T cell responses compared to a control whole cell lysate (WCL)-based vaccine. Prophylactic AMCNP vaccination provided protection from AML cell challenge in vivo. In a model of consolidation therapy, vaccination with AMCNPs after chemotherapy improved long-lasting immunity and significantly prolonged survival after AML re-challenge compared to WCL vaccination controls. These results demonstrate that AMCNPs are a novel and feasible approach for the development of effective AML vaccination immunotherapies.

## Materials and methods

### Plasmids

The MIP-OVA plasmid was created using overlap PCR; the murine *Cadm1* signaling peptide was cloned upstream of the mouse *Cadm1* transmembrane domain followed by a fusion with the full-length chicken *OVA* cDNA, which was then cloned into the MSCV-IRES- puromycin^R^ (MIP) plasmid [34] using bglll restriction enzyme sites. The *Cadm1* and *OVA* cDNA templates were from the pcDNA3-HA-*Cadm1* and the pODpCAGGS plasmid [35].

### Equalization of WCL vaccines

WCL was prepared by five freeze‐thaw cycles of liquid nitrogen followed by 10 min at 37 °C. The amount of WCL used was normalized by the amount of Na + /K + ‐ATPase protein, a characteristic membrane protein, compared with AMCNPs as determined by dot blotting (anti-ATP1A1 rabbit antibody, GenScript Biotech, Piscataway, NJ). Equivalent concentrations of CpG ODN 1826 (Integrated DNA Technologies, Coralville, IA) was added compared to AMCNP-encapsulated CpG.

### Dendritic cell maturation

For in vitro experiments, 1.5 × 10^6^ bone marrow-derived dendritic cells (BMDCs) were plated into 12-well suspension plates in BMDC growth media. Cells were pulsed for 2 h at a concentration of 1.4 mg/ml with C1498-OVA AMCNPs, equivalent C1498-OVA WCL vaccine, or equivalent CpG plus 1 μg/ml OVA SIINFEKL peptide (InvivoGen, San Diego, CA), then washed twice with fresh media. After 48 h of additional culture, cells were then collected in PBS with 1 mM EDTA and washed twice in PBS with 1% bovine serum albumin (Thermo Fisher Scientific). Cells were stained as indicated (Supplementary Table 1). For in vivo experiments, 50 µL of 25 mg/ml of AMCNPs, equivalent WCL vaccine, or mock treatment, were inoculated subcutaneously into each hock [36] of 8–12-week-old C57BL/6J mice (The Jackson Laboratory, Bar Harbor, ME). After 24 h, the popliteal lymph nodes of the mice were collected and manually dissociated in 500 µl dissociation buffer [Dulbecco’s PBS with calcium and magnesium (Gibco, Waltham, MA), 1 mg/ml collagenase D (Roche, Basel, Switzerland), and 1 mg/ml DNase I grade II (Roche)]. Cells were stained as indicated (Supplementary Table 1).

### B3Z OVA presentation assays

The B3Z CD8^+^ T cell hybridoma cell line is specific for OVA and expresses β-galactosidase under control of the IL-2 promoter [37]. For presentation assays, 1 × 10^4^ C1498-MIP or C1498-OVA cells were co-incubated with either 5 × 10^4^ (high) or 1 × 10^4^ (low) B3Z cells for 16 h at 37 °C, 5% CO_2_. For cross-presentation assays, 1 × 10^4^ DC2.4 cells or 5 × 10^4^ BMDCs were seeded into each well of a 96-well plate overnight. Serial dilutions beginning with 20 µl per well of 50 mg/ml AMCNPs or equivalent WCL vaccine were added for up to 4 h at 37 °C, 5% CO_2_. The cells were washed twice with PBS, fixed in 100 µl of cold PBS + 1% formaldehyde (Thermo Fisher Scientific) for 5 min, and then washed three times with 37 °C RPMI. B3Z cells (5 × 10^3^ per well) were incubated with the fixed APCs for 16 h at 37 °C, 5% CO_2_. Plates were washed twice with PBS, and 100 μl LacZ lysis buffer [0.13% NP-40, 9 mM MgCl_2_, 0.3 mM chlorophenol red-β-d-galactopyranoside (Roche) in PBS] was added for up to 4 h at 37 °C. Absorbance was measured at 570–650 nm.

### Ex vivo T cell functional assay

8–12-week-old C57BL/6J mice (The Jackson Laboratory) were vaccinated with 50 μl of 25 mg/ml of AMCNPs or equivalent WCL vaccine subcutaneously via the hock on days 0, 2, and 4. On day 10, spleens were extracted and mechanically dissociated into single cell suspensions. Red blood cell lysis was performed by resuspending the cell mixture in ice cold ACK buffer (0.1 mM Na_2_EDTA, 10 mM KHCO_3_, 150 mM NH_4_Cl) for 5 min followed by washing with ice cold PBS and passage through a 40-μM cell strainer (Thermo Fisher Scientific). 5 × 10^6^ splenocytes were then plated and cultured in six-well suspension plates in BMDC growth media with 20 ng/ml GM-CSF and 1 μg/ml of either OVA SIINFEKL (InvivoGen) or WT1 RMFPNAPYL (MBL international, Woburn, MA) peptide. After 5 or 7 days of ex vivo culture, respectively, the supernatant was collected and analyzed for interferon-γ (IFN-γ) using ELISA kits (Biolegend, San Diego, CA). At 7 days of ex vivo culture, the splenocytes were stained as indicated (Supplementary Table 1) and used for flow cytometry analysis.

### AMCNP consolidation vaccination

8–12-week-old C57BL/6J mice (The Jackson Laboratory) were challenged with 1 × 10^5^ C1498 cells via intravenous inoculation. 100 µl of 20 mg/ml cytarabine was administered via intraperitoneal injection on days 1, 2, 3, 4, and 5. Additionally, 30 µL of 2 mg/ml doxorubicin was administered via intraperitoneal injection on days 2, 3, and 4. The mice were housed with water containing 125 mg/l of ciprofloxacin and 20 g/l sucrose for the first 2 weeks of the experiment. The mice were vaccinated on days 26, 33, and 40 subcutaneously in both hocks with 50 µl of 25 mg/ml C1498 AMCNPs, equivalent amounts of C1498 WCL vaccine, or mock treatment. Mice were monitored with signs of morbidity as the endpoint. For the overall survival experiment, surviving mice were re-challenged on day 163 with 2 × 10^6^ C1498 cells and monitored for signs of morbidity. For the C1498-eGFP experiment, the surviving mice were re-challenged on day 72 with 2 × 10^6^ C1498-eGFP cells and all mice were euthanized, once the first mouse reached the endpoint, on day 93.

### Flow cytometry

Flow cytometry and cell sorting was performed as previously described [38]. The antibody staining schemes are listed (Table. S1). Analysis were performed using FlowJo software version 7.6.5. For calculating total events, flow cytometry samples containing either a known amount of peripheral blood (PB) or 3 × 10^6^ live splenocytes were run in their entirety and observed events were then normalized to 1 ml of PB or the total number of live splenocytes collected from each spleen.

### Statistical analyses

All statistical analyses were performed using GraphPad Prism Software (Version 8.4.2). The specific tests used are documented in the corresponding figure legend. The number of independent experiments is indicated in the figures, data is presented as mean values, and error bars represent standard deviation. All *t*-tests were two-tailed. *P* values are denoted as follows: ns *p* > 0.05, *p < 0.05, **p < 0.01, ***p < 0.001.

## RESULTS

### Production of AMCNPs for vaccination immunotherapy

**Fig. 1:**
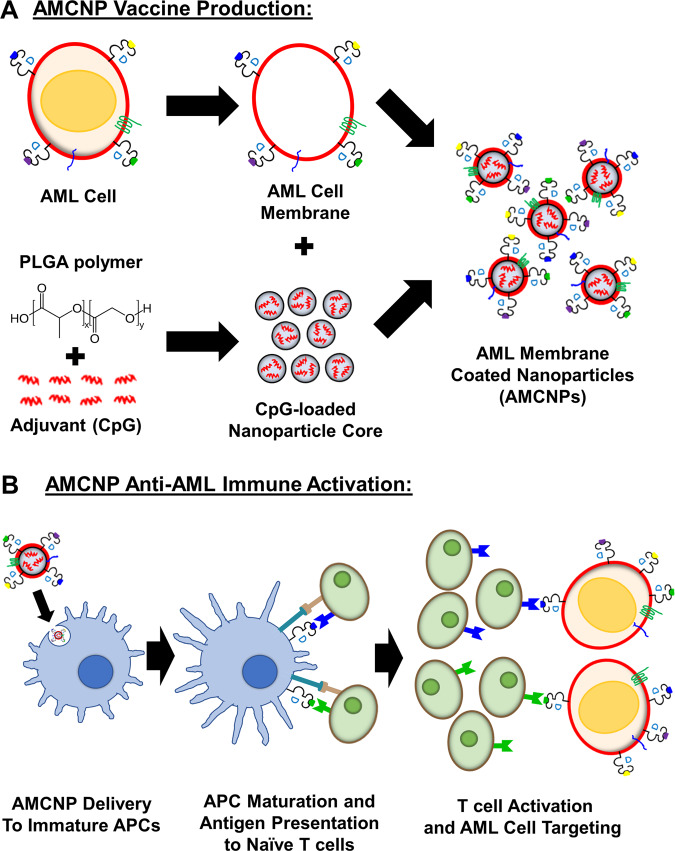
Schematic of AML membrane-coated nanoparticles (AMCNPs) production and anti-leukemic vaccination. **A** The immunostimulatory adjuvant, CpG oligodeoxynucleotide 1826, was encapsulated into biodegradable poly (lactic-*co*-glycolic acid) (PLGA) polymer nanoparticle cores (small gray spheres) via a double emulsion process. Through sonication, CpG-loaded nanoparticle cores were coated with isolated AML cell membrane (red circle), including membrane-associated MHC-I-presented antigens (blue, green, purple, and yellow dots), to form AMCNPs. **B** Delivery of AMCNPs to immature APCs (blue cell) stimulates maturation and AML-associated antigen presentation. The mature APCs (blue cell) present AML antigens and co-stimulatory molecules to naive T cells (green cells), resulting in activation and proliferation of T cells specific for different AML antigens (blue, green). Activated T cells (green cells) can initiate AML cell death, after detecting the MHC-I-presented antigens on AML cells (red cells).

To improve neoantigen vaccination strategies for AML, we examined a recently developed antigen presentation approach with AMCNPs containing immunostimulatory adjuvants [31–33]. The AMCNP strategy is designed to promote AML-specific immune responses by delivering nanoparticles (NPs) carrying multiple unidentified AML cell membrane-associated antigens and immunostimulatory adjuvants to APCs (Fig. 1). Through sonication, isolated membrane material is used to coat poly (lactic-*co*-glycolic acid) (PLGA) NP cores, which are synthesized using a double emulsion process. The final particles exhibit a core–shell structure uniformly coated with leukemia cell membrane [33]. A key advantage of AMCNPs is the capacity to package immune-stimulatory adjuvants within the NP core [33]. We used CpG oligodeoxynucleotide 1826, a Toll-like receptor 9 agonist, as a well-characterized and potent adjuvant in the current study (Fig. 1B). TLR agonists have been extensively used in vaccine immunotherapy due to their role in activating immune responses by stimulating APCs to upregulate co-stimulatory factors, secrete inflammatory cytokines, present antigens to T cells, and activate tumor-specific T cells [14].

**Fig. 2:**
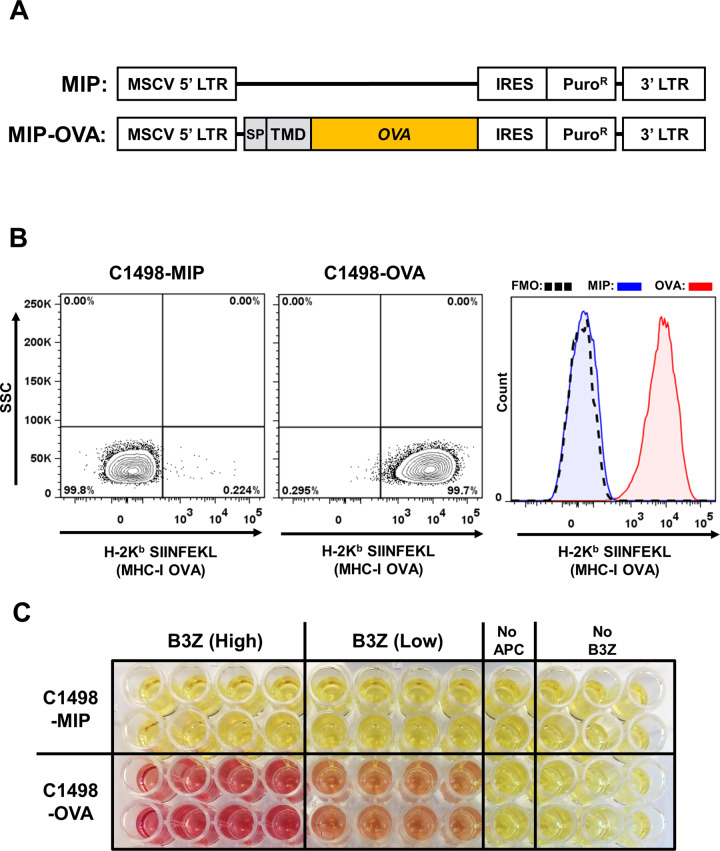
Characterization of C1498-OVA cell line. **A** MIP vector and MIP-OVA retrovirus constructs used in generation of C1498-MIP and C1498-OVA cell lines. The murine *Cadm1* signal peptide (SP) and transmembrane domain (TMD) were cloned 5’ to full-length chicken ovalbumin into the MIP vector. **B** Representative flow cytometry plots and histogram of C1498-MIP and C1498-OVA cell lines stained with antibodies against MHC class I-presented OVA peptide 257–264 (H-2K^b^:SIINFEKL), which demonstrate OVA antigen presentation in C1498-OVA cells compared to C1498-MIP or fluorescence minus one (FMO) negative control staining. **C** OVA-specific CD8^+^ T cell (B3Z) lacZ activation assay. C1498-MIP or C1498-OVA cells were incubated with B3Z CD8^+^ T cell hybridoma reporter cells, in which OVA-specific T cell receptor activation drives lacZ expression. Representative image demonstrating OVA-specific T cell activation (red color) in B3Z lysates, as assayed with the β-gal substrate chlorophenol red-β-galactoside (CPRG).

To test the efficacy of the AMCNP vaccine platform, the syngeneic C1498 murine AML cell line was employed. C1498 cells are derived from an AML that developed spontaneously in a C57BL/6 (H-2^b^) mouse [39]. The C1498 model has been used previously to examine various immunotherapeutic strategies [40–42]. We first used membrane-bound ovalbumin (OVA) as a model membrane-associated leukemic antigen for our study. To maximize OVA antigen expression on C1498 cells, sequences corresponding to the murine *Cadm1* signal peptide and transmembrane domain, along with full-length OVA, were cloned into the MSCV-IRES-puromycin^R^ (MIP) retroviral vector to generate MIP-OVA (Fig. 2A). MIP and MIP-OVA retrovirus-transduced C1498 cells are referred to as C1498-MIP and C1498-OVA cells, respectively. Presentation of OVA-derived peptides in the context of MHC class I molecules (H-2K^b^:SIINFEKL) on C1498-OVA cells was confirmed by flow cytometry (Fig. 2B). OVA-specific presentation by the C1498-OVA cell line was also confirmed through beta-galactosidase (lacZ) B3Z T cell activation assays [37], in which an OVA-specific CD8^+^ T cell activation lacZ reporter line (B3Z) was activated after co-culture with C1498-OVA cells but not C1498-MIP cells (Fig. 2C). Following intravenous inoculation of C1498-OVA cells, C57BL/6 mice uniformly succumbed, although at a later time point when compared to mice challenged with C1498 parental cells (C1498) (Fig. S1). The enhanced survival of mice challenged with C1498-OVA cells suggests that this cell line is more immunogenic than C1498 cells, likely due to the expression and presentation of the OVA antigen. Together, these results confirm that the C1498-OVA cell line expresses and presents OVA-derived antigens.

**Fig. 3:**
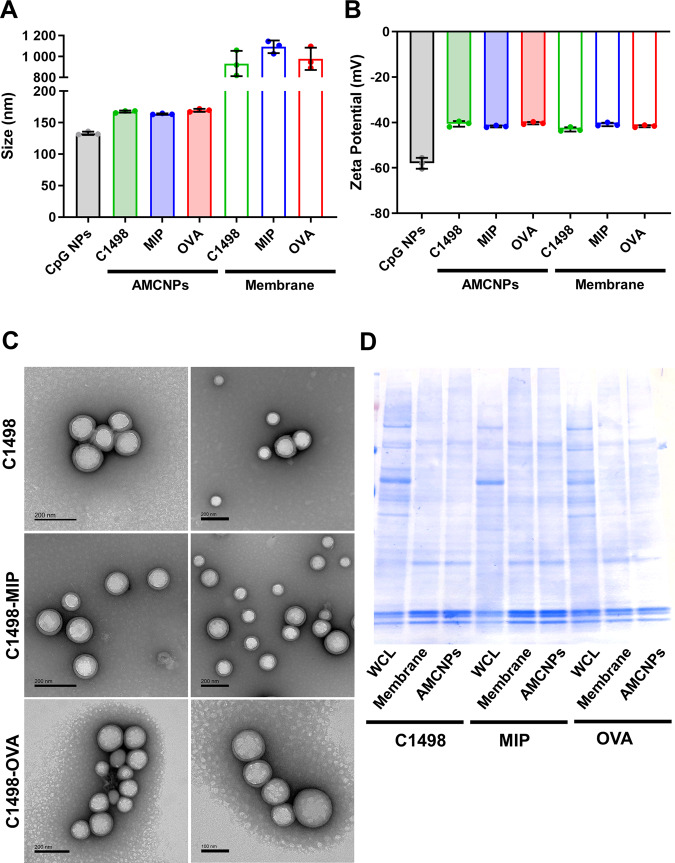
Characterization of AMCNPs. Uncoated nanoparticles (CpG NP), AMCNPs (C1498, C1498-MIP, and C1498-OVA), and isolated membrane material (C1498, C1498-MIP, and C1498-OVA) were analyzed for size (**A**) and zeta potential (**B**) through dynamic light scattering analysis. **C** Representative transmission electron microscopy images of C1498, C1498-MIP, and C1498-OVA AMCNPs. **D** Coomassie blue staining of whole cell lysates, isolated membrane material, and AMCNPs from C1498, C1498-MIP, and C1498-OVA cells.

AMCNPs were generated using membrane material from C1498-OVA, C1498-MIP, and C1498 cells. AMCNPs were loaded with CpG at roughly 100 pmol CpG/mg NP. Dynamic light scattering measurements were used to assess the size and zeta potential of the PLGA NP cores, AMCNPs, and isolated membrane material. AMCNPs showed slightly increased size compared to uncoated PLGA NPs (~165 nm vs 133 nm), consistent with successful membrane coating (Fig. 3A). As expected, the size of AMCNPs was far smaller than the corresponding isolated membrane material (~165 nm vs 1 µm) (Fig. 3A). Zeta potential is an important measurement of the effective electric charge on the NP surface. AMCNPs demonstrated increased zeta potential compared to uncoated PLGA NPs (~−40 mV vs −58 mV), consistent with successful membrane coating, as membrane material is less negatively charged than the PLGA NP core (~−40 mV vs −58 mV) (Fig. 3B). C1498 AMCNPs displayed a core-shell structure with uniform coating when observed through transmission electron microscopy (Fig. 3C). Furthermore, AMCNPs retained a very similar composition of membrane proteins when assessed by polyacrylamide gel electrophoresis protein separation (Fig. 3D). Together, these results confirmed our ability to successfully generate AMCNPs coated with leukemia cell-derived membrane material.

### APCs effectively acquire AMCNPs

**Fig. 4:**
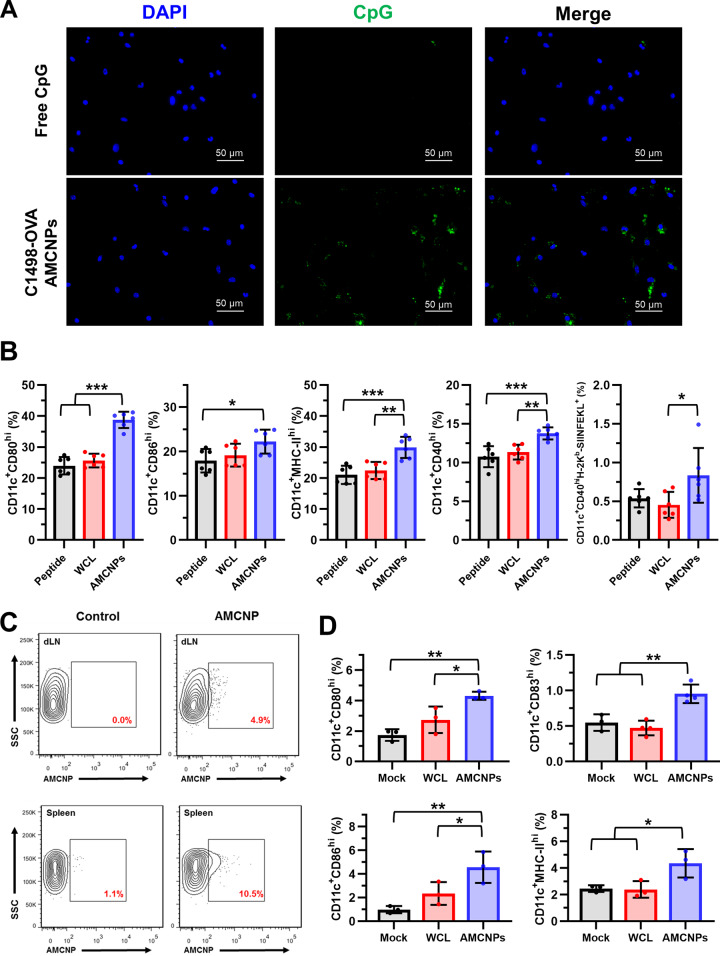
AMCNPs are taken up by APCs efficiently, stimulate maturation, and promote antigen presentation. **A** BMDCs were pulsed for 30 min with free dye-labeled CpG or equivalent C1498-OVA AMCNPs with encapsulated dye-labeled CpG. Representative images show cellular DNA staining by DAPI (blue), labeled CpG (green), and merged. **B** BMDCs were pulsed for 2 h with C1498-OVA AMCNPs, equivalent C1498-OVA whole cell lysate (WCL) vaccine, or OVA SIINFEKL peptide with CpG. 48 h post-pulsing, CD11c^+^ BMDCs were gated for high expression of the activation markers CD40, CD80, CD86, and MHC-II. Activated CD11c^+^CD40^hi^ BMDCs were further gated for MHC-I presentation of OVA (H-2K^b^:SIINFEKL). Data is presented as the mean percentage of total live BMDCs. **C** Labeled C1498 AMCNPs or mock controls were injected into C57BL/6 mice via the hock. 24 h post-injection, CD11c^+^ cells in the draining lymph node (dLN) and spleen were examined for presence of labeled C1498 AMCNPs. Representative flow cytometry plots are shown. **D** Mice received mock, C1498-OVA WCL, or equivalent C1498-OVA AMCNP vaccination. 24 h post-vaccination, CD11c^+^ cells in the dLN were gated for high expression of CD80, CD83, CD86, and MHC-II. Data is presented as mean percentage of total live cells. Significance was determined using one-way ANOVA with a post-hoc test using the Holm-Šídák method.

In order to stimulate effective immune responses, AMCNPs must first be acquired by APCs. Therefore, we examined the ability of AMCNPs to be captured by APCs by pulsing BMDCs with either dye-labeled CpG at a final concentration of 100 nM or equivalent C1498-OVA AMCNPs encapsulated with dye-labeled CpG. We observed efficient acquisition of labeled AMCNPs in BMDCs, where a detectable signal was initially observed after 30 min of pulsing (Figs. 4A and S2), which became readily visualized after 24 h (Fig. S2). In comparison to AMCNPs, BMDC acquisition of free CpG was far less detectable at both time points (Figs. 4A and S2). We also observed efficient acquisition of labeled C1498-OVA AMCNPs by the DC2.4 mouse DC cell line (Fig. S3A).

DC maturation is promoted through the engagement of pattern-recognition receptors, such as TLRs [43], with pathogen-associated molecular patterns, such as CpG [43, 44]. Thus, we next examined the ability of AMCNPs to promote BMDC maturation and antigen presentation by pulsing BMDCs for 2 h with 100 nM of either C1498-OVA AMCNPs, equivalent amounts (based on membrane material) of C1498-OVA WCL plus CpG (WCL vaccine), or CpG plus 1 μg/ml OVA SIINFEKL peptide (peptide vaccine). We observed enhanced maturation and activation of BMDCs pulsed with C1498-OVA AMCNPs as evidenced by the increased frequency of CD11c+ cells with high expression of the co-stimulatory molecules CD40, CD80, CD86, and MHC-II (Fig. 4B). Furthermore, the frequency of activated CD11c^+^CD40^hi^ BMDCs presenting the SIINFEKL peptide in the context of MHC-I (H-2K^b^:SIINFEKL) was significantly greater when pulsed with C1498-OVA AMCNPs, versus pulsing with either the WCL vaccine or the peptide vaccine (Fig. 4B).

To test the delivery of AMCNPs to APCs in vivo, fluorescently labeled AMCNPs were inoculated subcutaneously via the hock [36] of C57BL/6 mice. Labeled AMCNPs could be detected within CD11c^+^ DCs in the draining lymph node and spleen (Fig. 4C). To test the effect of the AMCNPs on in vivo DC maturation, C57BL/6 mice were inoculated subcutaneously into each hock [36] with 50 µL of 25 mg/ml of C1498-OVA AMCNPs, equivalent C1498-OVA WCL vaccine, or mock treatment. After 24 h, C1498-OVA AMCNP vaccination significantly increased DC maturation within the draining lymph node compared to vaccination with the C1498-OVA WCL vaccine or the mock control, as evidenced by increased frequency of CD11c^+^ cells with high expression of CD80, CD83, CD86, and MHC-II (Fig. 4D). Similar results were observed using C1498 AMCNP vaccination (Fig. S3B), suggesting that the promotion of DC maturation is not dependent upon the OVA antigen. Collectively, these results demonstrate that AMCNPs are efficiently delivered to APCs, induce maturation, and stimulate antigen presentation.

### AMCNPs enhance CD8^+^ T cell activation

**Fig. 5:**
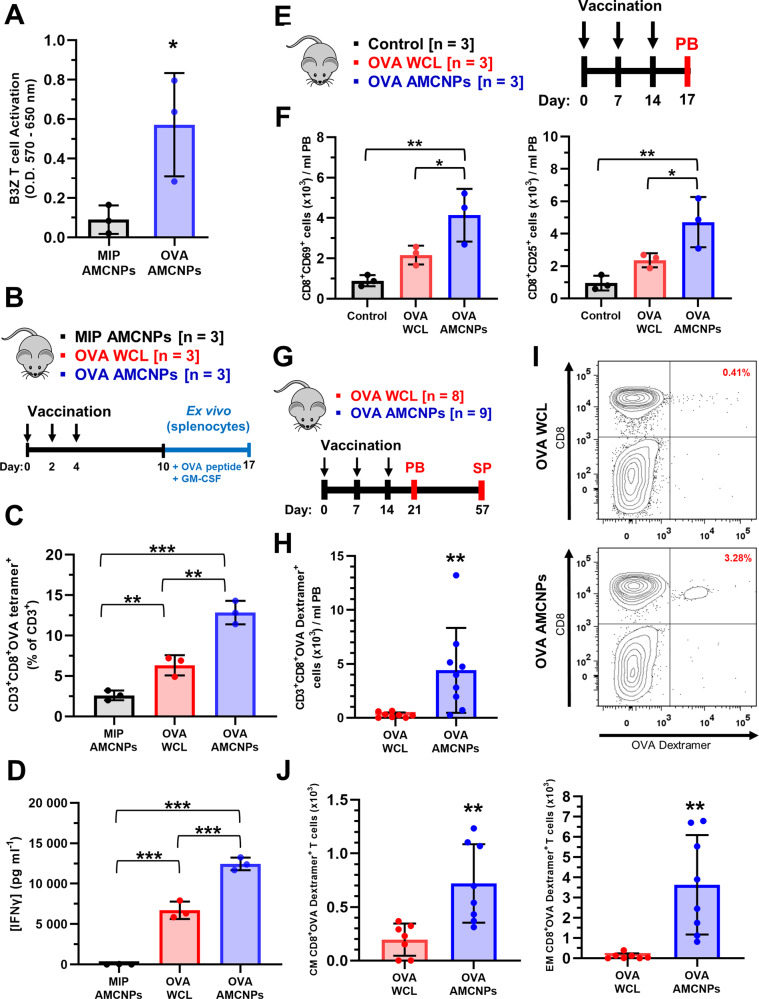
AMCNPs enhance antigen-specific T cell activation. **A** BMDCs were pulsed with C1498-MIP AMCNPs or C1498-OVA AMCNPs before co-culture with B3Z CD8^+^ T cells. OVA-specific B3Z CD8^+^ T cell activation was measured by a CPRG assay. Data shown as mean optical density at 570–650 nm from three experiments. Significance was determined by unpaired *t*-test. **B**–**D** Mice were vaccinated 3 times as indicated, with C1498-MIP AMCNPs, equivalent C1498-OVA WCL vaccine, or C1498-OVA AMCNPs; splenocytes were collected and re-stimulated ex vivo with OVA SIINFEKL peptide for 7 days (*n* = 3). **C** OVA-specific T cell expansion was measured by H-2K^b^:SIINFEKL tetramer staining of CD3^+^CD8^+^ T cells. Data is presented as the mean frequency of CD3^+^CD8^+^OVA tetramer^+^ cells among CD3^+^ cells. **D** The concentration of secreted IFN-γ was measured by ELISA. **C**, **D** Significance was determined using one-way ANOVA with a post-hoc test using the Holm-Šídák method. **E**, **F** Mice were vaccinated 3 times, as indicated, with C1498-OVA AMCNPs (*n* = 3), equivalent C1498-OVA WCL vaccine (*n* = 3), or mock vaccination control (*n* = 3). Total number of CD69^+^ or CD25^+^ CD8^+^ T cells among peripheral blood (PB) mononuclear cells was determined by flow cytometry on day 17 and normalized to 1 ml of PB. Significance was determined using one-way ANOVA with a post-hoc test using the Holm-Šídák method. **G**–**J** Mice were vaccinated 3 times, as indicated, with C1498-OVA AMCNPs (*n* = 9) or equivalent C1498-OVA WCL vaccines (*n* = 8). **H**, **I** OVA-specific T cell expansion was determined through staining with H-2K^b^:SIINFEKL dextramer (OVA-dextramer) of PB mononuclear cells on day 21. **H** Total CD3^+^CD8^+^OVA dextramer^+^ events observed were normalized to 1 ml of PB and adjusted for background staining by subtracting the average number of events in unvaccinated controls (*n* = 5). Significance was determined using one-way ANOVA with a post-hoc test using the Holm-Šídák method. **I** Representative flow cytometry plots of CD3^+^ gated live cells used to quantify CD3^+^CD8^+^OVA dextramer^+^ events are shown. **J** OVA-specific central memory (CM, CD62L^hi^CD44^hi^CD8^+^OVA dextramer^+^) and effector memory (EM, CD62L^low^CD44^hi^CD8^+^OVA dextramer^+^) expansion was determined through flow cytometry of live splenocytes on day 57. Total events observed were normalized to the total number of live splenocytes collected and adjusted for background staining by subtracting the average number of events in unvaccinated controls (*n* = 5). Significance was determined using one-way ANOVA with a post-hoc test using the Holm-Šídák method.

We next examined the ability of AMCNP-pulsed APCs to promote antigen-specific CD8^+^ T cell activation. BMDCs and DC2.4 cells were pulsed with C1498-OVA AMCNPs or C1498-MIP AMCNPs, and OVA-specific T cell activation was measured using B3Z OVA T cell activation assays [37]. C1498-OVA AMCNPs significantly stimulated OVA-specific T cell activation, whereas the C1498-MIP AMCNPs did not (Figs. 5A and S3C). These results suggest that antigens contained within the AMCNP are presented by APCs to stimulate specific T cell responses.

To test the efficacy of AMCNPs in vivo, C57BL/6 mice were vaccinated on days 0, 2, and 4 via subcutaneous inoculation into each hock [36] with 50 µl of 25 mg/ml C1498-MIP AMCNPs, C1498-OVA AMCNPs, or equivalent C1498-OVA WCL vaccine; their splenocytes were collected on day 10 and re-stimulated ex vivo with OVA-derived peptide (SIINFEKL) until day 17 (Fig. 5B). A significantly higher frequency of ex vivo-stimulated splenic CD8^+^ T cells from C1498-OVA AMCNP-vaccinated mice were labeled by H-2K^b^:SIINFEKL tetramers, compared to C1498-OVA or C1498-MIP AMCNP-vaccinated mice, suggesting that C1498-OVA AMCNP vaccination drives the expansion of antigen-specific CD8^+^ T cells (Fig. 5C). Furthermore, ex vivo-stimulated splenocytes from C1498-OVA AMCNP-vaccinated mice secreted significantly higher concentrations of IFN-γ compared to those vaccinated with C1498-MIP AMCNPs or C1498-OVA WCL vaccines (Fig. 5D).

We next sought to directly examine activation and OVA-specific T cell expansion in vivo. Mice were vaccinated with C1498-OVA AMCNPs or equivalent C1498-OVA WCL vaccine on days 0, 7, and 14 (Fig. 5E, F). On day 17, we observed significantly increased numbers of CD8^+^CD69^+^ and CD8^+^CD25^+^ T cells among the PB mononuclear cells of AMCNP-vaccinated mice compared with WCL-vaccinated or control mice (Fig. 5F); suggesting that AMCNP vaccination stimulates activation of CD8^+^ T cells. OVA-specific T cell expansion was then assessed by H-2K^b^:SIINFEKL dextramer (OVA dextramer) staining on day 21 (Fig. 5G–I). Significantly higher numbers of CD3^+^CD8^+^ OVA dextramer^+^ T cells were found in AMCNP-vaccinated mice compared with WCL-vaccinated mice (Fig. 5H). To examine OVA-specific memory CD8^+^ T cells, splenocytes were collected from the mice on day 57 (Fig. 5H, J). Significantly higher numbers of OVA-specific central memory (CD62L^hi^CD44^hi^CD8^+^OVA dextramer^+^) and effector memory (CD62L^low^CD44^hi^CD8^+^OVA dextramer^+^) CD8^+^ T cells were observed in AMCNP-vaccinated mice compared with WCL-vaccinated mice (Fig. 5J).

We next sought to demonstrate AMCNP mediated immune responses against a known leukemia-associated antigen, Wilms’ tumor 1 (WT1). WT1 is a well characterized leukemia-associated antigen that has been targeted in various AML vaccination therapies [30, 45]. Because C1498 cells do not express WT1 [46], we generated a WT1 expressing C1498 cell line (C1498-WT1) (Fig. S4A). WT1 expression in the C1498-WT1 line was confirmed by western blotting (Fig. S4B). To examine WT1 specific T cell expansion in vivo, mice were vaccinated with C1498-WT1 AMCNPs or equivalent C1498-WT1 WCL vaccine on days 0, 7, and 14 (Fig. S4C). Using H-2D^b^:RMFPNAPYL dextramer (WT1 dextramer) staining of PB mononuclear cells on day 21, we observed significantly higher numbers of CD3^+^CD8^+^ WT1 dextramer^+^ T cells in the AMCNP-vaccinated mice compared with WCL-vaccinated mice (Fig. S4C). Additionally, splenocytes from AMCNP-vaccinated mice secreted significantly higher concentrations of IFN-γ compared to WCL-vaccinated mice, after ex vivo-stimulation with WT1 peptide (Fig. S4D). Collectively, these data suggest that in vivo vaccination with AMCNPs mediates potent CD8^+^ T cell immunity against leukemia-specific and leukemia-associated antigens.

### AMCNP vaccination protects against AML cell challenge

We next sought to determine if immune responses elicited by AMCNPs provided protective anti-leukemic immunity. To first confirm that AMCNP vaccination could elicit anti-leukemic immunity against AML cells which expressed a known antigen, we prophylactically vaccinated C57BL/6 mice with three rounds of 50 µl of 25 mg/ml C1498-OVA AMCNPs (*n* = 5) or mock treatment with equivalent CpG (*n* = 5) before challenging them with 1 × 10^5^ C1498-OVA cells (Fig. S5A). All C1498-OVA AMCNP-treated mice survived more than 21 weeks after challenge with C1498-OVA cells, whereas none of the control mice survived past 19 weeks (median survival 8.7 weeks) (Fig. S5B). These results suggested that AMCNP vaccination provides prophylactic anti-leukemic immunity in a context in which AML-specific antigens are being expressed.

**Fig. 6:**
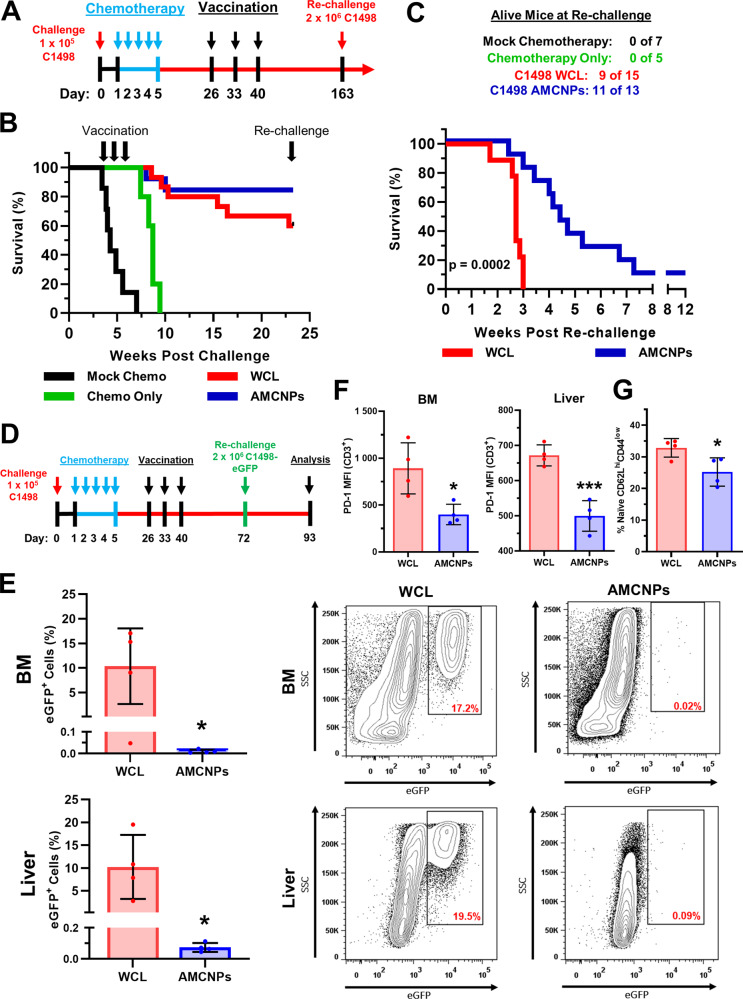
Post-remission AMCNP vaccination promotes long-lasting anti-leukemic immunity and survival benefit. **A** Mice were challenged with 1 × 10^5^ C1498 cells, followed by either cytarabine and doxorubicin chemotherapy or mock chemotherapy (*n* = 7). **B** Mice were then vaccinated at 26-, 33-, and 40-days post-challenge with C1498 AMCNPs (*n* = 13) or equivalent C1498 whole cell lysate vaccine (WCL) (*n* = 15). Unvaccinated “chemotherapy only” mice were used as controls (*n* = 5). **C** Surviving mice were re-challenged at day 163 with 2 × 10^6^ C1498 cells. Kaplan–Meier survival plots are shown with significance determined by the Mantel–Cox test. **D**–**G** Mice were challenged with 1 × 10^5^ C1498 cells, followed by cytarabine and doxorubicin chemotherapy. Mice were then vaccinated at 26-, 33-, and 40-days post-challenge with C1498 AMCNPs (*n* = 4) or equivalent C1498 WCL vaccine (*n* = 4). Mice were re-challenged at day 72 with 2 × 10^6^ C1498-eGFP cells and analyzed at day 93. **E** Frequency of eGFP^+^ cells among mononuclear cells isolated from the bone marrow (BM) or liver of vaccinated mice. Representative flow plots are shown. **F** MFI of PD-1 expression among BM and liver CD3^+^ T cells from AMCNP-vaccinated and WCL-vaccinated mice. **G** Splenic CD3^+^CD8^+^ T cells from AMCNP-vaccinated and WCL-vaccinated mice were analyzed for the frequency of naive T cells (CD62L^hi^CD44^low^). Significance was determined using unpaired *t*-tests.

We further sought to test C1498 AMCNPs in a more therapeutically relevant remission model, in which vaccination occurs after initial induction therapy with conventional chemotherapeutic drugs. C57BL/6 mice challenged with 1 × 10^5^ C1498 cells were subsequently treated with clinically relevant AML chemotherapy drugs [5], cytarabine and doxorubicin, prior to vaccination (Fig. 6A). Cytarabine and doxorubicin treatment significantly prolonged survival in unvaccinated, “chemotherapy only” mice (*n* = 5, median survival 8.7 weeks) compared to mock chemotherapy controls (*n* = 7, median survival 4.3 weeks) (Fig. 6B). Mice were vaccinated with C1498 AMCNPs (*n* = 13) or C1498 WCL vaccine (*n* = 15) at 4-, 5-, and 6-weeks post-chemotherapy (Fig. 6A). Both AMCNP and WCL vaccine groups had significantly prolonged survival compared to the chemotherapy only group (median survival 8.7 weeks) by week 21 (Fig. 6B). The AMCNP-vaccinated group had a higher survival rate (85%) than the WCL vaccine group (60%) at 21 weeks post-C1498 challenge. However, this difference was not significantly greater due to the high survivability of both groups (Fig. 6B). To observe the longer-term effect of AMCNPs, we re-challenged surviving mice with 2 × 10^6^ C1498 cells 21 weeks after the initial challenge. After re-challenge, the AMCNP-vaccinated group showed significant survival benefit (median survival 4.4 weeks post-re-challenge) compared to the WCL-vaccinated mice (median survival 2.7 weeks post-re-challenge) (Fig. 6C). To further examine the anti-leukemic effect of AMCNPs in our remission model, additional C1498 AMCNP-vaccinated (*n* = 4) and C1498 WCL-vaccinated (*n* = 4) mice underwent the same course of C1498 cell challenge, chemotherapy, and vaccination; however, these mice were re-challenged with 2 × 10^6^ C1498 cells that stably expressed enhanced GFP (C1498-eGFP) 32 days after the final vaccination (Fig. 6D). The AMCNP-vaccinated mice had a significantly lower percentage of C1498-eGFP cells in the bone marrow and liver compared to WCL-vaccinated mice at 21 days post-re-challenge (Figs. 6E and S6). The WCL vaccination mice had significantly higher expression levels of the programmed cell death protein 1 (PD-1) inhibitory receptor within both BM and liver CD3^+^ T lymphocyte populations, compared to AMCNP-vaccinated mice (Fig. 6F). Interestingly, the AMCNP-vaccinated mice showed a significant decrease in the frequency of naive CD8^+^ T cells (CD62L^hi^ CD44^low^) (Figs. 6G and S7), suggesting that AMCNP-vaccinated mice have an increased proportion of antigen-exposed CD8^+^ T cells. Taken together, these results suggest that AMCNP vaccination during remission improves long-lasting immunity and significantly delays disease progression upon re-challenge, compared to WCL vaccination.

## Discussion

The lack of well-tolerated and effective consolidation therapy strategies is a major gap in the current AML standard of care, due to the significant morbidity and imperfect prevention of relapse associated with allogeneic HSCT [5, 6, 47]. This gap remains despite recent progress in benefiting patients with various subtypes of AML through targeted therapies, such as hypomethylating agents, immune checkpoint inhibitors, FLT3 inhibitors, and isocitrate dehydrogenase inhibitors [7, 8]. AML vaccination therapies have proven to be generally tolerable and to consistently promote leukemia antigen-associated T cell immunity; thus, displaying their therapeutic potential [10, 45]. Additionally, the efficacy of AML vaccination may be further improved when combined with targeted therapies, such as hypomethylating agents [48] and immune checkpoint inhibitors [17, 49]. We developed an AMCNP vaccination therapy that is personalized, multi-antigenic, and can deliver both membrane-associated leukemic antigens as well as immunostimulatory adjuvants to professional APCs in vivo.

In our C1498 AML relapse model, consolidation therapy using AMCNPs provided a significant benefit to overall survival compared to WCL vaccines. Importantly, this benefit was observed after re-challenge 4 months post-vaccination using a 20-fold increase in the number of C1498 leukemia cells than in the initial challenge. Thus, AMCNPs induced a long-term benefit, even without additional therapeutic interventions, such as immune checkpoint inhibitors. We observed far fewer leukemic cells in the bone marrow and liver of AMCNP-vaccinated versus WCL-vaccinated mice in our C1498-eGFP re-challenge model. Correspondingly, we observed increased expression levels of PD-1 in the T cells of WCL-vaccinated mice, which is associated with AML immune-suppression [50, 51] and is a mechanism of immune evasion in C1498 cells [52], suggesting that AMCNP vaccination might be beneficial in preventing immune evasion during AML relapse. AMCNP vaccination was also associated with a significant decrease in naive CD8^+^ T lymphocytes within the spleen, suggesting that an increased proportion of CD8^+^ T cells in the AMCNP-vaccinated mice have been exposed to leukemic antigens. Collectively, we demonstrate that AMCNP vaccination enhanced anti-leukemic immunity.

AML is a heterogeneous disease, in which individual patients often have varying leukemic clonal populations. A single suitable protein target that is present in all AMLs has yet to be identified and likely does not exist. AML blasts between and within patients may present clonal AML-specific neoantigens, due to random mutation [53]. Thus, there is great need for AML immunotherapies that are both fully personalized and multi-antigenic to overcome these challenges. Indeed, the feasibility of recurrent neoantigen immunotherapy in AML has recently been demonstrated [15, 16]. However, personalized vaccines that rely on identifying patient-specific neoantigens are technically challenging, time-consuming, and expensive. Our AMCNP vaccination strategy is multi-antigenic, fully personalized, and obviates the need for neoantigen identification. Therefore, AMCNP vaccination therapy is suitable for AML.

The AMCNP vaccine strategy is amenable to further development and refinement for potential clinical applications. A key advantage of the AMCNP strategy is the colocalization of both antigen and adjuvant, ensuring that individual APCs receive both, which promotes enhanced antigen-specific immune responses [12, 54, 55]. We show that CpG-encapsulated AMCNPs are efficiently acquired by APCs, increase co-stimulatory signal expression (CD80, CD86, CD83, CD40, and MHC-II), and promote leukemic antigen presentation. Of note, empty PLGA particles themselves are unable to induce APC activation and cytotoxic T cell responses [33, 55, 56]. Furthermore, PLGA particles are widely used [57] and have already been approved by the US FDA for drug delivery. While CpG has often been used as an adjuvant in preclinical models [14], there are many additional immunostimulatory adjuvants [58]. Indeed, great efforts have been made to identify which adjuvants, or combinations of adjuvants, are best in clinical settings [58]. AMCNPs can potentially be packaged with a variety of different adjuvants; thus, this strategy is adaptable to future advances in clinical adjuvant use.

In summary, our AMCNP vaccine is multi-antigenic, personalized, co-localizes adjuvant with antigens, and is efficiently delivered to APCs. We demonstrate the successful development of the AMCNPs. We further show that AMCNPs outperform a control WCL vaccine in activating AML-specific immune responses and providing long-term anti-leukemic survival benefit when used as a consolidation therapy. Thus, AMCNPs are a promising platform that can be further developed and refined as an AML vaccination immunotherapy.

## Supplementary information

Supplementary Material
