# Supplementary material for: Acute myeloid leukemia cell membrane-coated nanoparticles for cancer vaccination immunotherapy
Source: Leukemia. 2021 Nov 29;36(4):994–1005. doi: 10.1038/s41375-021-01432-w (PMC8979812; doi:10.1038/s41375-021-01432-w)
Supplement: Supplementary file 1 — Supplementary Material [file 41375_2021_1432_MOESM1_ESM.docx]

**SUPPLEMENTARY METHODS**

**Plasmids**

The MIP-WT1 plasmid was created using PCR; the full-length murine *Wt1* cDNA was cloned into the MSCV-IRES-puromycin^R^ (MIP) plasmid^1^ using bglll restriction enzyme sites. The murine *Wt1* MGC premier full length cDNA clone (transOMIC, Huntsville, AL) was used as the template for cloning.

**Cell culture**

HEK293T cells were purchased from ATCC. C1498 cells were a kind gift from Dr. Shujun Liu (The Hormel Institute, University of Minnesota). B3Z T-cell hybridoma cells were a kind gift from Dr. Nilabh Shastri (University of California, Berkeley). HEK293T, C1498, and associated lines were cultured in DMEM media supplemented with 10% FBS (HyClone, Logan, UT) and 100 U/ml penicillin-streptomycin (Gibco, Waltham, MA). C1498-MIP, C1498-OVA, and C1498-WT1 cells were maintained in 0.5 µg/ml puromycin (InvivoGen, San Diego, CA). B3Z and DC2.4 cells were cultured in RPMI media supplemented with 10% FBS and 100 U/ml penicillin-streptomycin. BMDCs were cultured in BMDC culture media consisting of RPMI media supplemented with 10% FBS, 50 µM β-mercaptoethanol (Gibco), 100 U/ml penicillin-streptomycin, and 20 ng/ml murine GM-CSF (Peprotech, Rocky Hill, NJ). All cells were maintained in a 37°C incubator with 5% CO_2_.

**Viral transduction**

For retroviral transduction experiments, retrovirus was produced in HEK293T cells. Transfections of HEK293T cells were conducted by combining 5 μg of MSCV-IRES-Puro^1^ (MIP), MIP-OVA (OVA), MIP-WT1 (WT1), or MSCV-IRES-eGFP^2^ (MIGR1) vectors with 5 μg of packaging vector (EcoPak) and 40 μl of 1 mg/ml polyethylenimine (Polysciences Inc, Warrington, PA) in 1 ml of Opti-MEM reduced serum medium (Gibco). Approximately 16 h post-transfection, the media was aspirated, cells were washed once in PBS, and 7 ml of fresh DMEM media supplemented with 10% fetal bovine serum and 100 U/ml penicillin-streptomycin was added to each plate. At 24 h after the media change, DMEM media containing retroviral particles was collected, passed through a 0.45-μm syringe filter, pooled, and supplemented with 4 μg/ml polybrene (Millipore Sigma, Burlington, MA). C1498 cells were resuspended in retroviral media at a concentration of approximately 0.5 × 10^6^ cells/ml and were transduced in 6-well plates by centrifugation (2,000 × *g*) for 3 h at 32°C in an Allegra X-12R centrifuge (Beckman Coulter, Brea, CA) on two consecutive days. At 24 h following the second transduction, C1498-MIP, C1498-WT1, and C1498-OVA cells were resuspended in fresh DMEM media supplemented with 10% fetal bovine serum, 100 U/ml penicillin-streptomycin (Gibco), and 1 μg/ml puromycin (Millipore Sigma). After 48 h of puromycin selection, cells were diluted and maintained at 0.5 μg/ml puromycin until future analysis. C1498-eGFP (C1498-MIGR1) cells were sorted for the top 25% of eGFP fluorescence and maintained in DMEM media supplemented with 10% fetal bovine serum and 100 U/ml penicillin-streptomycin.

**Membrane derivation**

C1498 cells and associated variants were grown in T175 suspension flasks (Genesee Scientific, El Cajon, CA) and harvested every 3 to 4 days. For cell harvesting, the cell suspension media was collected, and any loosely adherent cells were detached with 1 mM EDTA (Corning, Corning, NY) in PBS (Corning). Cells were spun down at 700 × *g* for 5 min and the packed pellet was resuspended in complete medium mixed 1:1 with cryopreservation media (Hyclone). The cells were stored in -20°C until enough harvests were accumulated. To derive the membrane, cell harvests were washed three times with a buffered solution containing 30 mM Tris-HCl pH 7.5 (Quality Biological, Gaithersburg, MD), 2.6% (w/v) sucrose (Millipore Sigma), and 4.1% (w/v) D-mannitol (Millipore Sigma) and then mechanically lysed twice with a Kinematica Polytron PT 10/35 probe homogenizer at 70% power for 15 s in a solution containing protease inhibitor (Millipore Sigma), phosphatase inhibitor (Millipore Sigma), and 500 µM ethylene glycol-bis(β-aminoethyl ether)-N,N,N′,N′-tetraacetic acid (Millipore Sigma). Intracellular contents were purified out by centrifuging the resulting homogenate at 10,000 × *g* for 25 min in an Optima XPN-80 ultracentrifuge (SW32 Ti, Beckman Coulter). The membrane in the supernatant was pelleted at 150,000 × *g* for 35 min and then buffer exchanged into a storage solution containing 200 µM ethylenediaminetetraacetic acid (EDTA, MP Biomedicals, Santa Ana, CA) in DNase free/RNase free water (Invitrogen, Carlsbad, CA). Finally, the membrane was resuspended, and the protein content was determined with a BCA protein assay kit (Pierce Biotechnology, Waltham, MA) according to manufacturer’s instructions. All membrane derivation procedures were performed on ice or at 4°C and the resulting membrane was stored at -20°C until use.

**Nanoparticle fabrication**

The nanoparticles were synthesized with a double emulsion technique as previously reported^3^. Briefly, 25 nmol of CpG ODN 1826 with a full phosphorothioate backbone and sequence 5′‐TCCATGACGTTCCTGACGTT‐3 (Integrated DNA Technologies, Coralville, IA) was dissolved in 200 mM Tris-HCl pH 8.0 (Corning) and mixed with 25 mg of 0.18 dl/g carboxyl-terminated 50:50 poly(lactic-*co­*-glycolic) acid (PLGA, LACTEL Absorbable Polymers) dissolved in dichloromethane (Thermo Fisher Scientific, Waltham, MA) to form the inner phase. The mixture was probe sonicated with a 150E sonic dismembrator (Thermo Fisher Scientific) at 70% power with a 2 s on/1 s off pulse for 1 min. Then an emulsified solution containing 100 µl of dichloromethane and 5 ml of 10 mM Tris-HCl pH 8.0 was added into the CpG–polymer mixture as the outer phase and sonicated with the same settings for 2 min. The final emulsion was added into 10 ml of 10 mM Tris-HCl pH 8.0 and magnetically stirred (Thermo Fisher Scientific) at 700 rpm for at least 2.5 h to evaporate the dichloromethane. The NPs were spun down at 21,100 × *g* for 8 min, consolidated into a single tube, and washed three times with 10 mM Tris-HCl pH 8.0 to remove free CpG. At the last wash, different types of membrane (C1498, C1498-MIP, C1498-WT1, or C1498-OVA) were used to resuspend the nanoparticles at a 1:10 membrane to polymer ratio, and the mixture was sonicated for 2 min with a FS30D bath sonicator (Thermo Fisher Scientific) in macro disposable cuvettes (BrandTech Scientific, Essex, CT). Excessive membrane was similarly washed out three times with 10 mM Tris-HCl pH 8.0 as before and the NPs were resuspended in 10% sucrose (Millipore Sigma, Burlington, MA) buffered with 5 mM Tris-HCl pH 7.5 and 200 µM EDTA in DNase free/RNase free water at a final concentration of 25 mg/ml. NPs were frozen in -20°C if they were not used on the same day. Fluorescently labeled NPs were prepared similarly by incorporating either 5’6-FAM-modified CpG (ex/em = 495/520 nm, Integrated DNA Technologies) or 1,1′‐dioctadecyl‐3,3,3′,3′‐tetramethylindodicarbocyanine, 4‐chlorobenzenesulfonate salt (DiD, ex/em = 644/663 nm, Biotium, Fremont, CA) at a 0.1% (w/w) ratio in the inner phase.

**Nanoparticle characterization**

Characterization of the nanoformulation was performed on particles resuspended at a final concentration of 5 mg/ml in DNase/RNase free water instead of the sucrose solution. The size and zeta potential of the nanoparticles were measured in a folded capillary zeta cell (Malvern Panalytical, Malvern, United Kingdom) using a Malvern ZEN3600 Zetasizer. Measurements for the membrane was similarly determined but at a final concentration of 500 µg/ml to reflect the coating ratio. The morphology of the nanoparticles was visualized via transmission electron microscope using a JEOL 1200 EX II transmission electron microscope. To prepare the imaging samples, the nanoparticle suspension was adsorbed onto a 400-mesh carbon film grid (Electron Microscopy Sciences, Hatfield, PA) for 10 min followed by three washes with distilled water for 2 min each. The grid was then negatively stained with 1% (w/v) uranyl acetate for 15 s and allowed to dry prior to imaging.

**Protein characterization**

C1498, C1498-MIP, and C1498-OVA whole cell lysates (WCL) were prepared by five freeze‐thaw cycles in liquid nitrogen followed by 10 min at 37°C. The protein concentration of the WCL, membrane, and AMCNP samples were determined in tube format with a BCA protein assay kit. All samples were diluted to a final concentration of 500 µg/ml and mixed with NuPAGE LDS sample buffer (Novex, Waltham, MA) at a 3 to 1 ratio. Protein samples were denatured at 70°C for 10 min in a Isotemp digital dry bath (Thermo Fisher Scientific) and loaded into a 12-well Bolt 4-12% bis-tris gel (Novex) along with the SeeBlue Plus2 pre-stained protein standard (Invitrogen, Waltham, MA). The proteins were separated at 165 V for 45 min in Bolt MOPS SDS running buffer (Novex) in the mini blot module system and the gel was stained with Instant Blue (Expedeon, Heidelberg, Germany) at room temperature for 1 h. Excessive dye was washed off in distilled water and images were taken the following morning.

**Western blot**

Primary antibodies used were anti-α-tubulin (1:10,000) antibody (12G10, Developmental Studies Hybridoma Bank, University of Iowa, Iowa City, IA) and anti-WT1 (1:1000) antibody (12609-1-AP, Protein Tech Group, Rosemont, IL). Licor (Lincoln, NE) IRDye 680RD goat anti-mouse IgG and IRDye 800CW goat anti-rabbit IgG secondary antibodies (1:10,000) were used for visualization on a LI-COR Odyssey Classic imager. Image analysis was performed using the LI-COR Application Software Version 3.0.

**Animals**

All animal protocols were approved by the UCSD Institutional Animal Care and Use Committee. Mice were housed and monitored in accordance with institutional guidelines.

**Leukocyte cell collection and isolation**

Peripheral blood was collected via submandibular vein bleeding^4^. Bone marrow cells were harvested by flushing two femurs per mouse with ice cold PBS using disposable syringes with 21-gauge needles. Spleen and liver leukocytes were harvested by physical tissue disruption and repeated pipetting in ice cold PBS. Red cell lysis was performed by resuspending cell mixture in ice cold ACK buffer (0.1 mM Na_2_EDTA, 10 mM KHCO_3_, 150 mM NH_4_Cl) for 5 min, followed by washing with ice cold PBS and passage through a 40-μM cell strainer (Thermo Fisher Scientific) to eliminate large tissue/cell clumps and debris. Live leukocytes were enriched via ficoll (Cytiva, Amersham, United Kingdom) gradient centrifugation. Viable cells were counted via trypan blue exclusion using a TC20 Automated Cell Counter (Bio-Rad Laboratories, Hercules, CA) to infer total cell numbers.

**Multimer Staining**

Prior to multimer staining, isolated cells were treated with 50 nM dasatanib^5^ (R&D Systems, Minneapolis, MN) for 30 min at 37°C and then Fc receptors were blocked for 10 minutes on ice. Tetramer (MBL international, Woburn, MA) or Dextramer (immudex, Copenhagen, Denmark) staining was performed according to manufacturer’s protocols. For tetramer staining, cells were washed twice prior to additional of cell surface antibody staining.

**Generating BMDCs**

Bone marrow cells were harvested by flushing two femurs per C57BL/6J mice (The Jackson Laboratory, Bar Harbor, ME) with ice cold PBS using disposable syringes with 21-gauge needles. Live cells were cultured in BMDC culture medium in untreated petri dishes at a density of 2 × 10^6^ cells per plate. After 3 days, an additional 10 ml of BMDC culture medium was added to each dish. On day 7, non-adherent and loosely adherent cells were removed. Purified mouse BMDCs were analyzed for purity by flow cytometry (Supplementary Table 1) before use in associated experiments.

**AMCNP acquisition by BMDCs**

BMDCs were seeded into 4-well Nunc chamber slides (Thermo Fisher Scientific) at a density of 1 × 10^5^ cells/well and allowed to adhere overnight. FAM-labeled CpG-encapsulated AMCNPs (C1498 or C1498-OVA) or equivalent amounts of free FAM-labeled CpG were added into each well at a final CpG concentration of 100 nM, either 24 h or 30 min before imaging. The media was discarded, and cells were washed three times with PBS to remove any free nanoparticles or FAM-labeled CpG. BMDCs were then fixed for 15 min at room temperature in 10% buffered formalin (Thermo Fisher Scientific) and washed three times as before. The chamber was finally removed, and the slide was mounted with VECTASHIELD mounting media with DAPI (Vector Laboratories, Burlingame, CA). Images were acquired with a Keyence BZ-X710 fluorescence microscope using a 40× objective lens equipped with the DAPI and GFP filters (UCSD Specialized Cancer Center Support P30 Grant 2P30CA023100).

**AMCNP acquisition by DC2.4 murine DC cell line**

DC2.4 were seeded into 24-well suspension plates (Genesee Scientific) at a density of 2.5 × 10^4^ cells/well and allowed to adhere overnight. At various times before analysis (24 h, 12 h, 6 h, 2 h, 1 h, 30 min, 15 min, and 0 min), DiD-labeled CpG-AMCNPs (C1498-MIP) were added into each well at a final CpG concentration of 250 nM. The media was collected, and any adhered cells were detached with 0.25% Trypsin-EDTA (Gibco). The cells were spun at 700 × *g* for 5 min and free nanoparticles were washed three times with 1% (w/v) bovine serum albumin (BSA, Millipore Sigma, Burlington, MA) in PBS. FITC-labeled Annexin V (Biolegend) was used to stain the cells at room temperature for 15 min as a viability dye and data was acquired with a BD FACSCanto II flow cytometer (BD Biosciences, San Jose, CA).

***In vivo* delivery**

To monitor nanoparticle trafficking *in vivo*, 50 µl of each nanoformulation was injected subcutaneously through the right hock of six-week-old C57BL/6NHsd mice (Envigo, Indianapolis, IN). Mice were euthanized 24 h later via carbon dioxide asphyxiation followed by cervical dislocation, and the organs were excised. All organs were mechanically homogenized with a pipette and processed into a single cell suspension. The spleens were filtered with a 70-µm mesh cell strainer (Thermo Fisher Scientific) and the draining lymph nodes were filtered with a 40-µm Flowmi cell strainer (Bel Art Products, Wayne, NJ). Red blood cells were lysed using a commercial RBC lysis buffer (Biolegend, San Diego, CA) before the cells were blocked with 1% BSA (Millipore Sigma) in PBS on ice for 15 min and with TruStain FcX PLUS anti-mouse CD16/32 antibody (Biolegend) for 10 min. Cells were stained as indicated (Supplementary Table 1) and analyzed by flow-cytometry.

**AMCNP prophylactic vaccination**

8- to 12-week-old C57BL/6J mice (The Jackson Laboratory) were vaccinated via subcutaneous hock injection with 50 µL of 25 mg/ml of C1498-OVA AMCNPs, or mock treatment with equivalent CpG, on days 0, 7, and 14. On day 21, mice were challenged with 5 × 10^5^ C1498-OVA cells via intravenous inoculation. Mice were monitored for signs of morbidity as an endpoint.

**SUPPLEMENTARY REFERENCES**

1. Peterson LF, Wang Y, Lo MC, Yan M, Kanbe E, Zhang DE. The multi-functional cellular adhesion molecule CD44 is regulated by the 8;21 chromosomal translocation. Leukemia. 2007;21(9):2010-9.

2. Pear WS, Miller JP, Xu L, Pui JC, Soffer B, Quackenbush RC, et al. Efficient and rapid induction of a chronic myelogenous leukemia-like myeloproliferative disease in mice receiving P210 bcr/abl-transduced bone marrow. Blood. 1998;92(10):3780-92.

3. Kroll AV, Fang RH, Jiang Y, Zhou J, Wei X, Yu CL, et al. Nanoparticulate Delivery of Cancer Cell Membrane Elicits Multiantigenic Antitumor Immunity. Adv Mater. 2017;29(47).

4. Golde WT, Gollobin P, Rodriguez LL. A rapid, simple, and humane method for submandibular bleeding of mice using a lancet. Lab Anim (NY). 2005;34(9):39-43.

5. Dolton G, Tungatt K, Lloyd A, Bianchi V, Theaker SM, Trimby A, et al. More tricks with tetramers: a practical guide to staining T cells with peptide-MHC multimers. Immunology. 2015;146(1):11-22.

**Supplementary Table 1. Flow-cytometry staining schemes.**

**
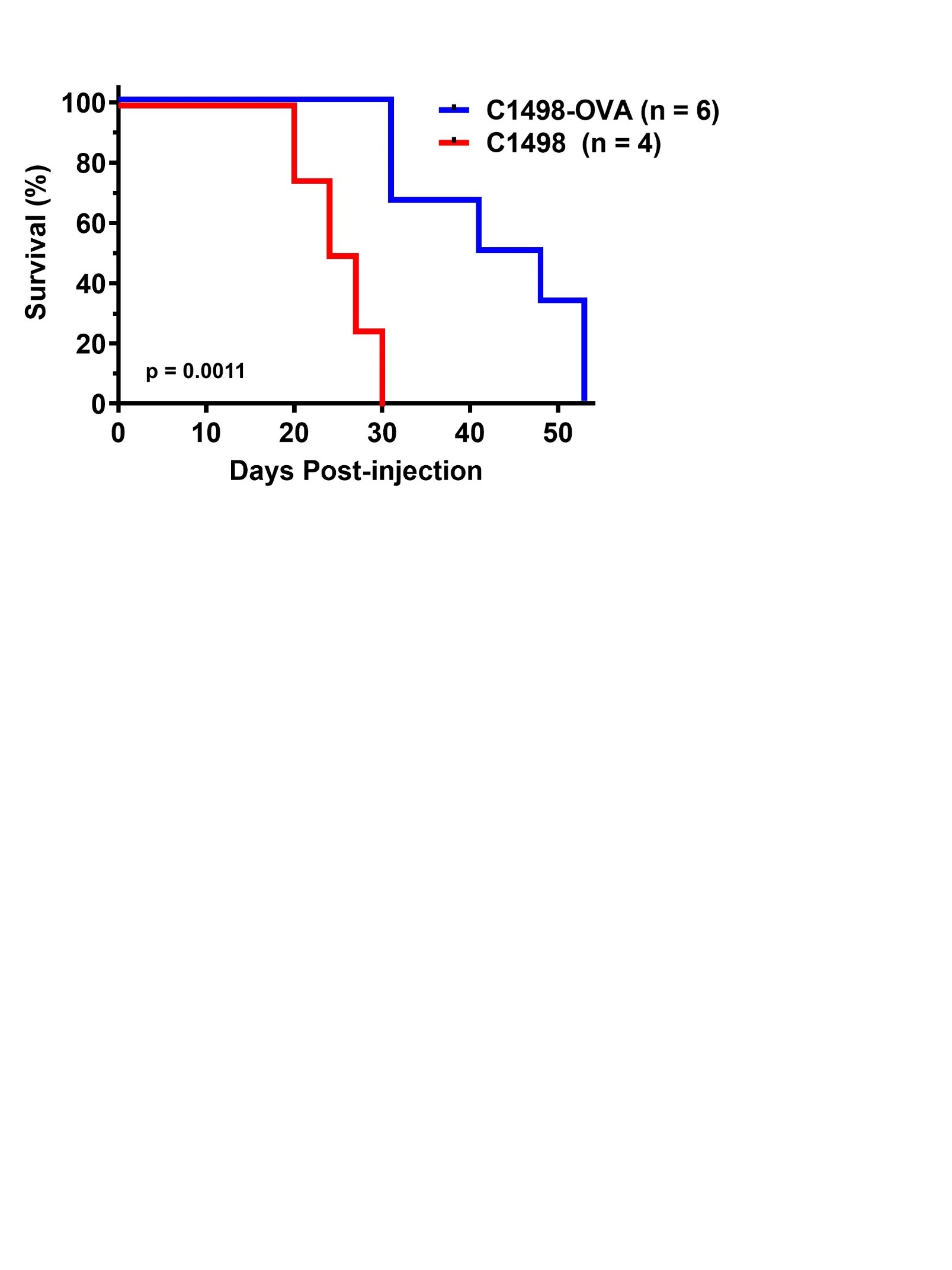
Supplementary Figure 1.** **C1498-OVA cells are leukemogenic.**

**Supplementary Figure 2. *In vitro* AMCNP acquisition by BMDCs.
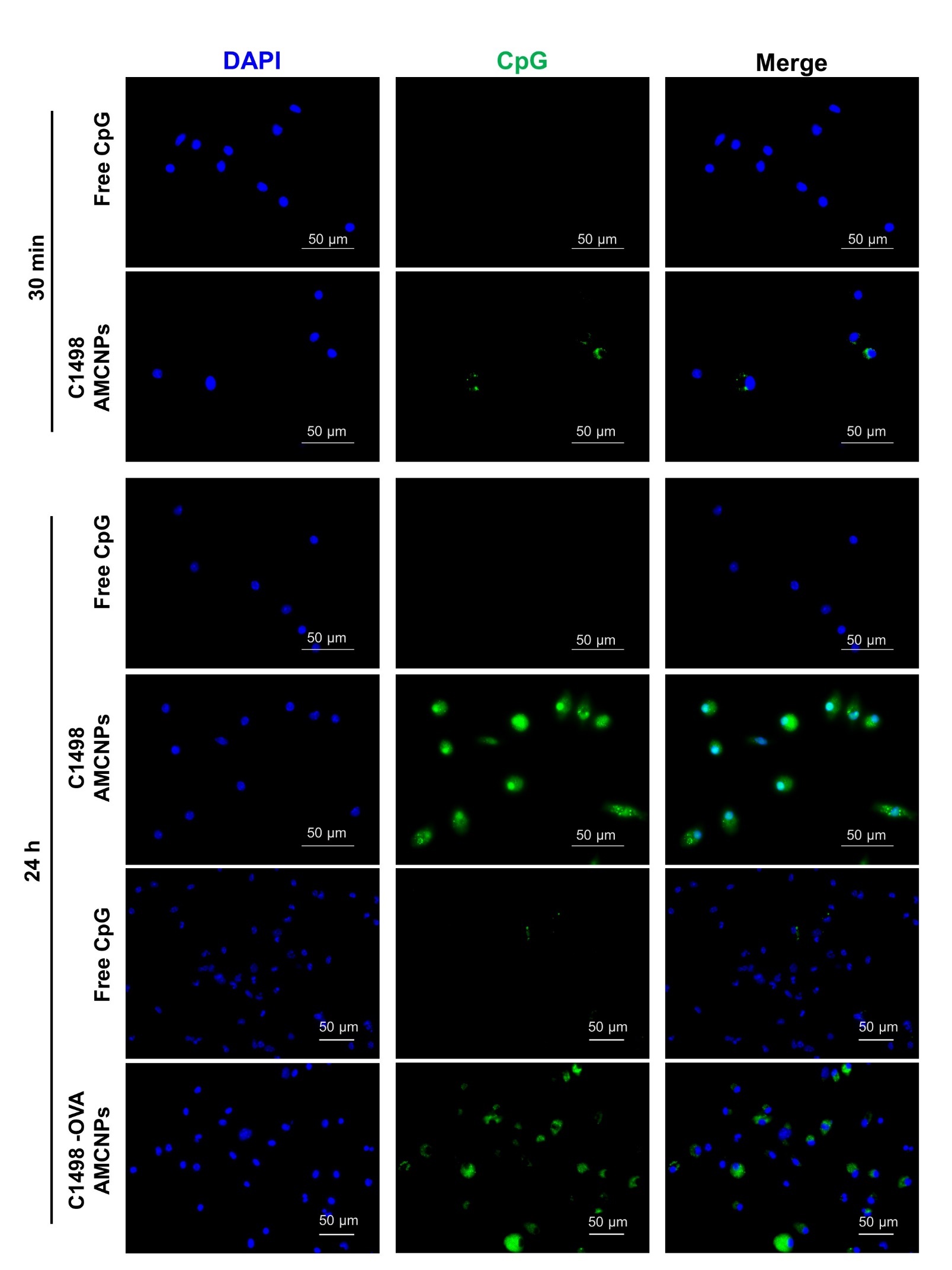
**

**
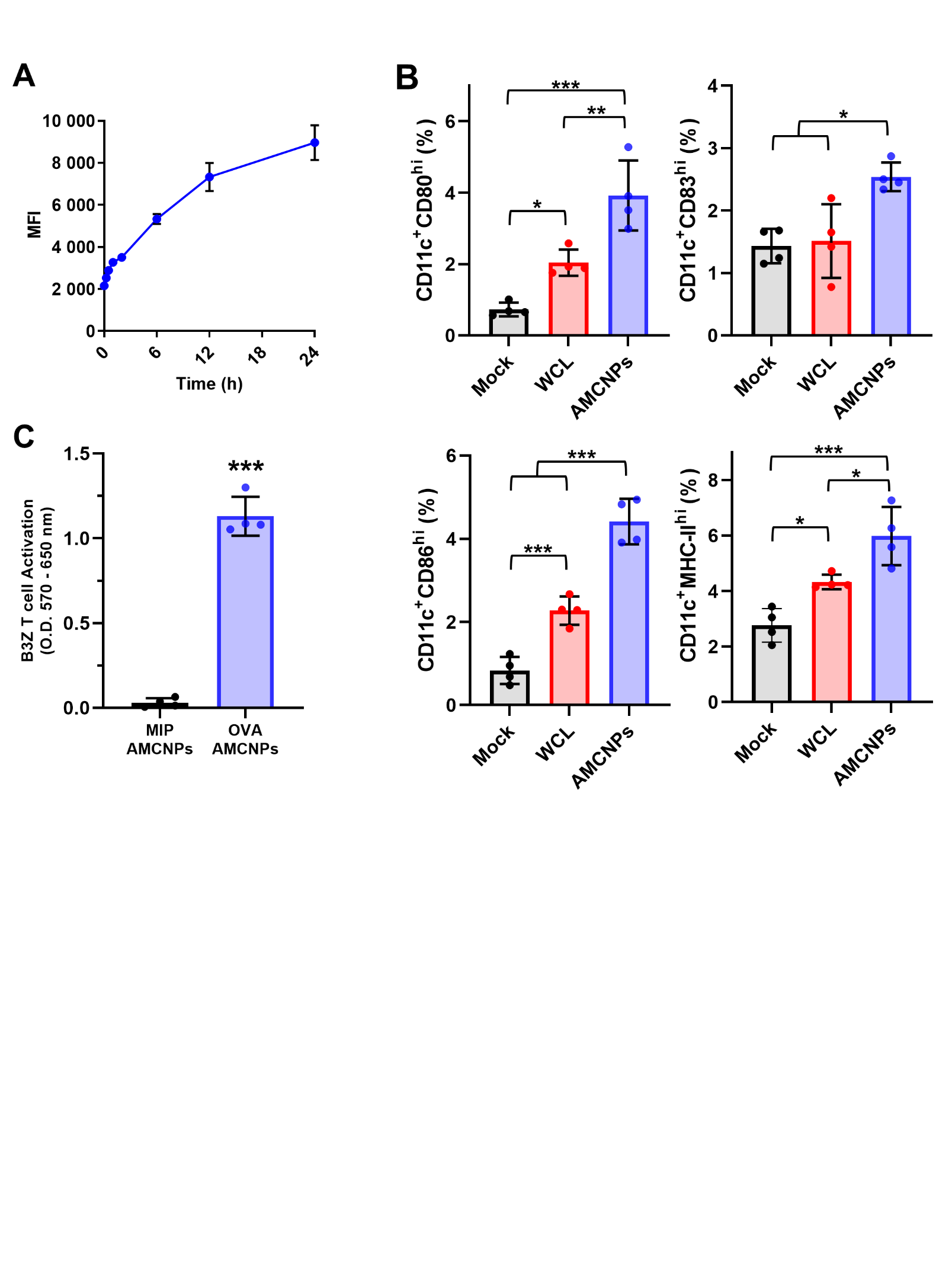
Supplementary Figure 3. Additional AMCNP acquisition, antigen presentation, and maturation data.**

**Supplementary Figure 4. AMCNPs enhance AML associated antigen T cell response.
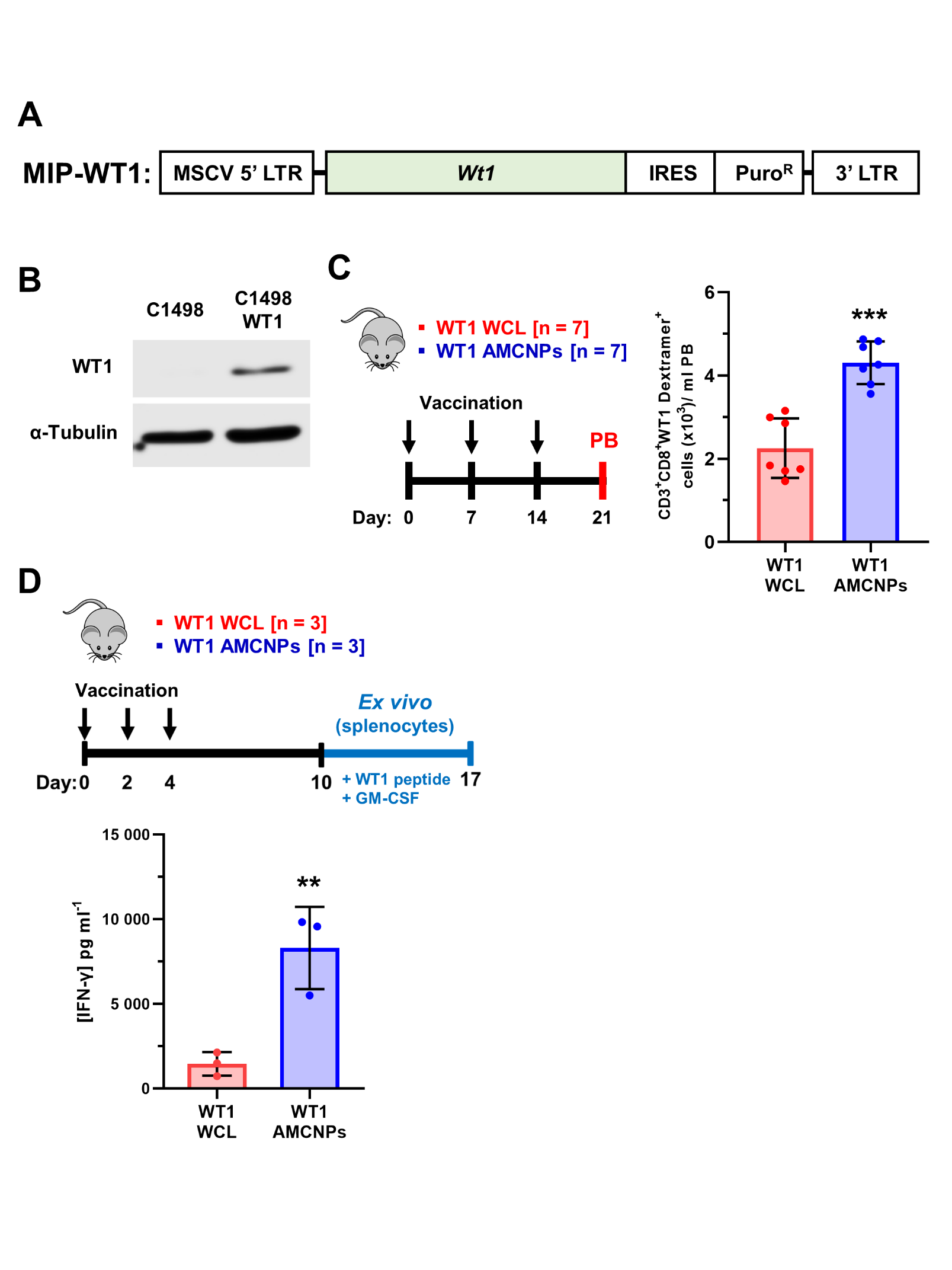
**

**Supplementary Figure 5. Prophylactic AMCNP vaccination protects against C1498-OVA AML challenge.
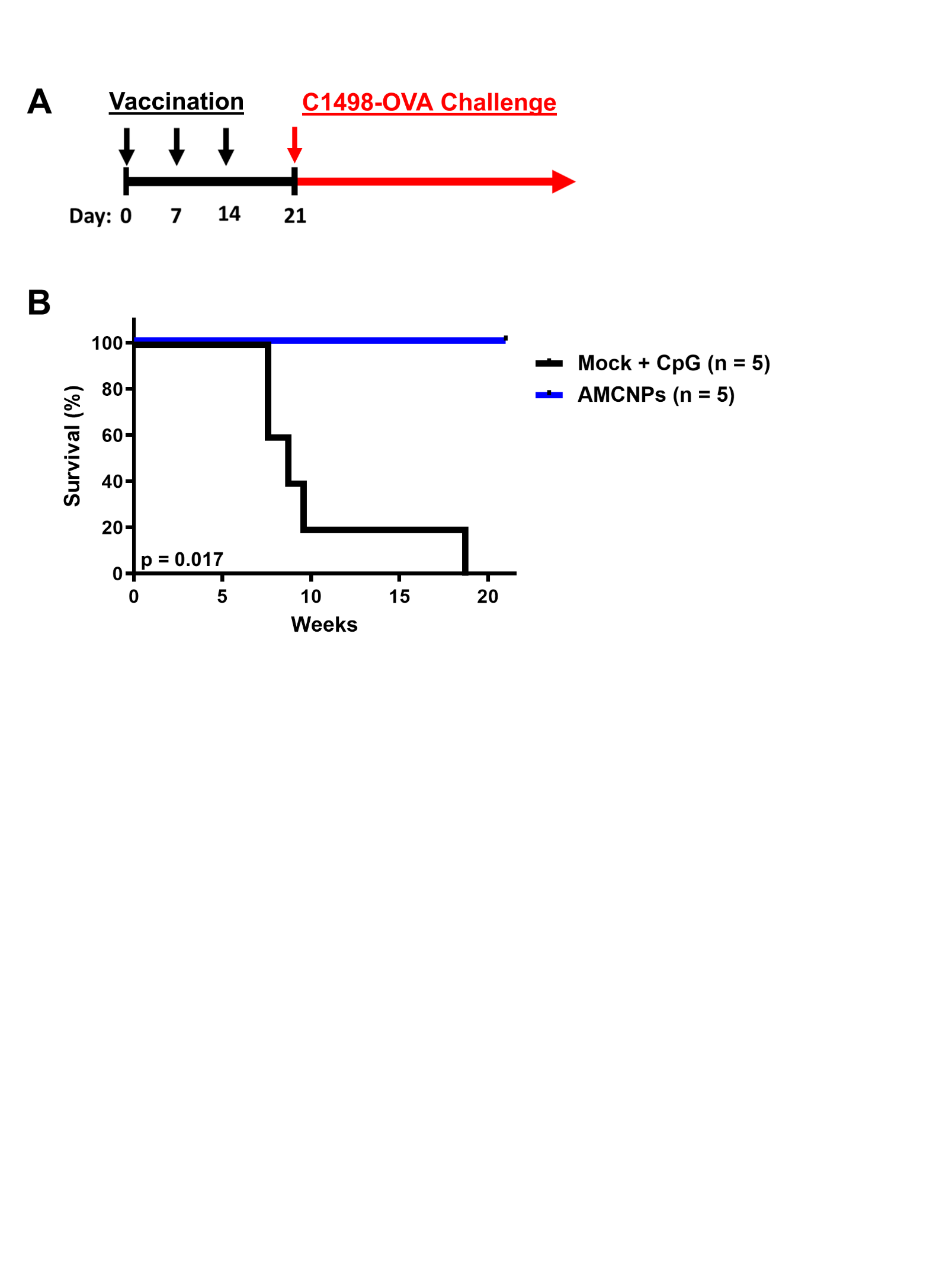
**

**Supplementary Figure 6. Flow cytometry gating of C1498-eGFP re-challenge cells.
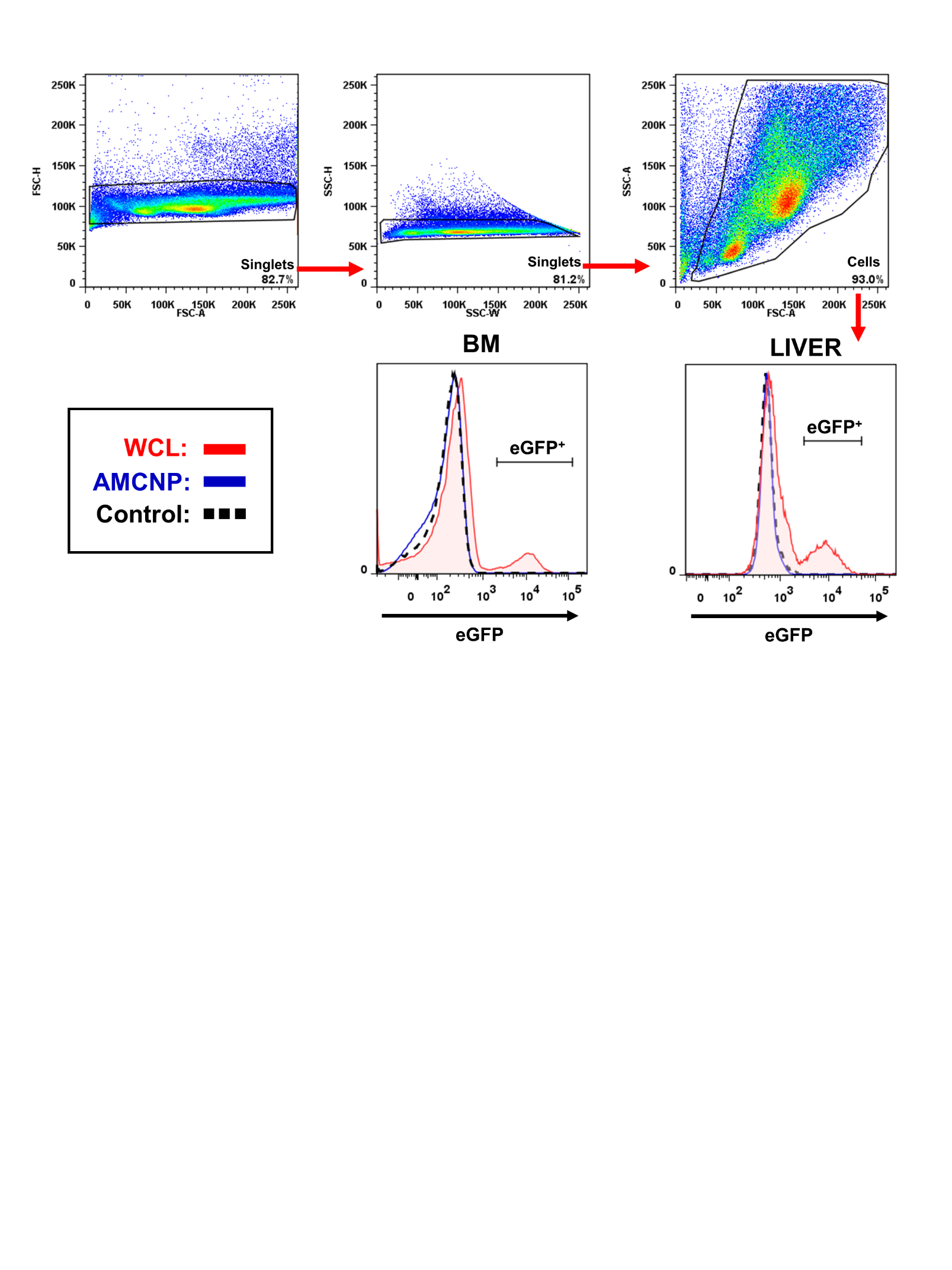
**

**Supplementary Figure 7. Flow cytometry for splenic CD3^+^CD8^+^ memory T cell subsets.
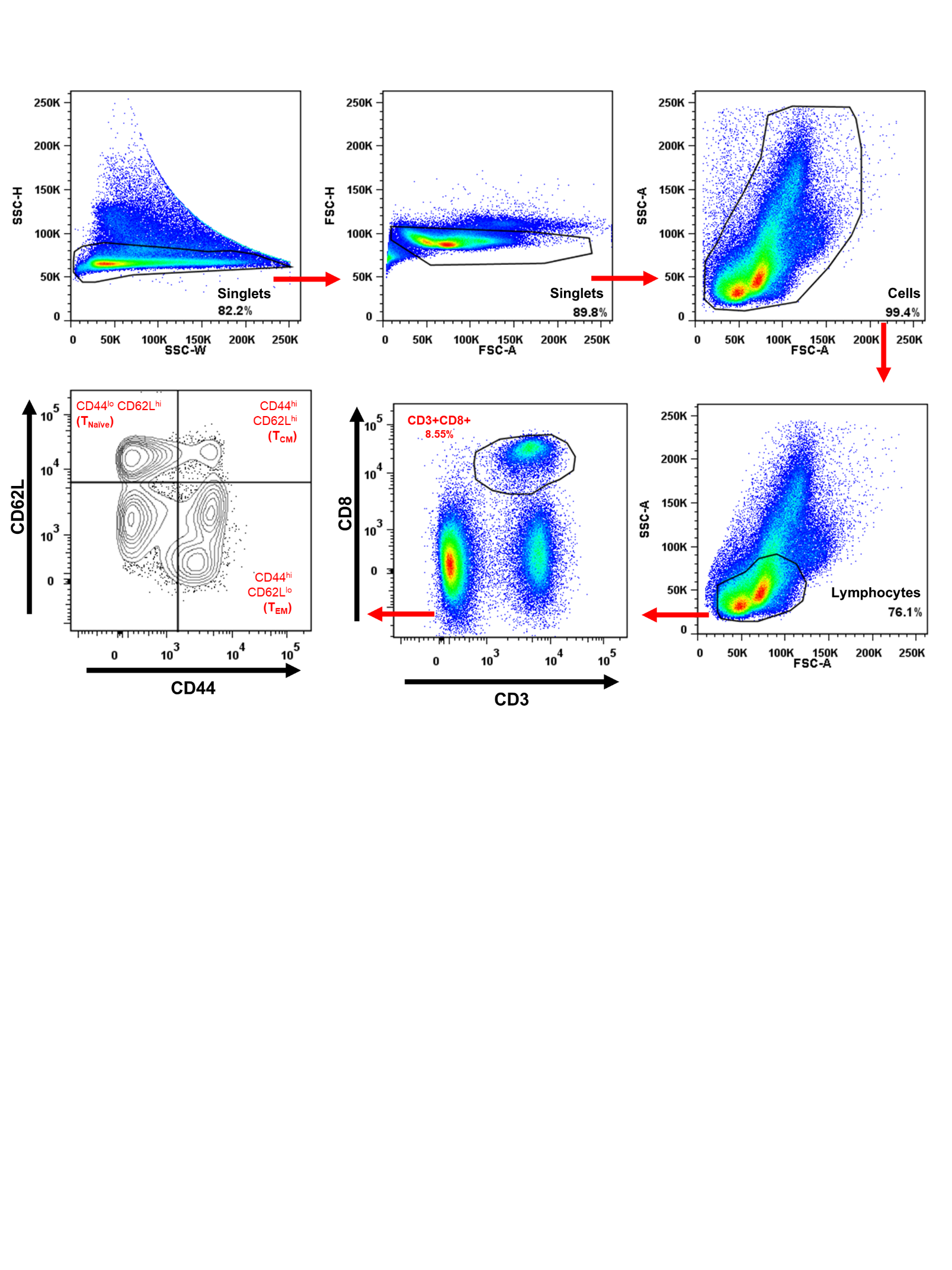
**

**SUPPLEMENTARY FIGURE LEGENDS**

**Supplementary Figure 1.** **C1498-OVA cells are leukemogenic.** Kaplan-Meier survival plot for C57BL/6 mice injected with 5 × 10^5^ C1498 (n = 4) or C1498-OVA cells (n = 6). Significance determined by the Mantel-Cox test.

**Supplementary Figure 2. *In vitro* AMCNP acquisition by BMDCs.** BMDCs were pulsed for 30 min or 24 h with free dye-labeled CpG, or equivalent C1498-OVA or C1498 AMCNPs with encapsulated dye-labeled CpG. Representative images show cellular DNA staining by DAPI (blue), dye-labeled CpG (green), and merged.

**Supplementary Figure 3. Additional AMCNP acquisition, antigen presentation, and maturation data.** (**A**) DC2.4 cells were pulsed with C1498-OVA AMCNPs with encapsulated dye-labeled CpG. AMCNP acquisition was detected through fluorescence over time. Data shown as mean fluorescence intensity (arbitrary units) from n = 3 biological replicates. (**B**) Mice received mock, C1498 WCL, or equivalent C1498 AMCNP vaccination. 24 h post-vaccination, CD11c^+^ cells in the dLN were gated for high expression of CD80, CD83, CD86, and MHC-II. Data is presented as mean percentage of total live cells. Significance was determined using one-way ANOVA with a post-hoc test using the Holm- Šídák method. (**C**) DC2.4 cells were pulsed with C1498-MIP AMCNPs or C1498-OVA AMCNPs before co-culture with B3Z T cells. OVA-specific B3Z T cell activation was measured by CPRG assay. Data shown as mean optical density at 570 - 650 nm. Significance was determined by unpaired t-test.

**Supplementary Figure 4. AMCNPs enhance AML associated antigen T cell response. (A)** MIP-WT1 retrovirus constructs used in generation of the C1498-WT1 cell line. The full length murine *Wt1* cDNA was cloned into the MIP vector. (**B**) WT1 expression was confirmed in C1498-WT1 cells by western blot. (**C**) Mice were vaccinated 3 times, as indicated, with C1498-WT1 AMCNPs (n = 7) or equivalent C1498-WT1 WCL vaccines (n = 7). WT1-specific T cell expansion was determined through staining with H-2D^b^: RMFPNAPYL dextramer (WT1-dextramer) on peripheral blood (PB) mononuclear cells on day 21. Total CD3^+^CD8^+^WT1-dextramer^+^ events observed were normalized to 1 ml of PB and adjusted for background staining by subtracting the average number of events in unvaccinated controls (n = 5). Significance was determined by unpaired t-test. (**D**) Mice were vaccinated 3 times as indicated, with C1498-WT1 AMCNPs or equivalent C1498-WT1 WCL vaccine; splenocytes were collected and re-stimulated *ex vivo* with WT1 RMFPNAPYL peptide for 7 days (n = 3), the concentration of secreted IFN-γ was measured by ELISA. Significance was determined by unpaired t-test.

**Supplementary Figure 5. Prophylactic AMCNP vaccination protects against C1498-OVA AML challenge. (A)** Mice were vaccinated on days 0, 7, and 14 with 50 µl of 25 mg/ml C1498-OVA AMCNPs (n = 5) or mock with equivalent CpG (n = 5). At day 21, mice were challenged with 1 × 10^5^ C1498-OVA cells. **(B)** Kaplan–Meier survival plot is shown with significance determined by the Mantel-Cox test.

**Supplementary Figure 6. Flow cytometry gating of C1498-eGFP re-challenge cells.** Bone marrow and liver mononuclear leukocytes were isolated from the mice used in Figure 6D. Representative flow plots and histograms showing gating strategy used to examine live eGFP^+^ cells from mice vaccinated with C1498 AMCNPs, C1498 whole cell lysate control vaccine (WCL), or healthy control mice (Control).

**Supplementary Figure 7. Flow cytometry for splenic CD3^+^CD8^+^ memory T cell subsets.** Splenic mononuclear cells were isolated from the mice used in Figure 6D. Representative gating of CD3^+^CD8^+^ CD62L^hi^ CD44^low^ naïve T cells used in Figure 6D.
